# ‘Today Was Probably One of the Most Challenging Workdays I’ve Ever Had’: Doing Remote Qualitative Research with Hospital Doctors During the COVID-19 Pandemic

**DOI:** 10.1177/10497323221106294

**Published:** 2022-06-07

**Authors:** Niamh Humphries, John-Paul Byrne, Jennifer Creese, Lorna McKee

**Affiliations:** 1 RCSI Graduate School of Healthcare Management, RCSI University of Medicine and Health Sciences, Dublin, Ireland; 2 Research Department, 8867 Royal College of Physicians of Ireland, Dublin, Ireland; 3 SAPPHIRE (Social Science Applied to Healthcare Improvement Research), 4488 University of Leicester, Leicester, UK; 4 1019 University of Aberdeen, Aberdeen, Scotland, UK

**Keywords:** health worker;, healthcare work environment;, qualitative;, ethnography;, reflexivity;, Ireland;, COVID-19 pandemic

## Abstract

In this article we outline how a team of qualitative researchers responded to the challenging circumstances of the COVID-19 pandemic, describing how we successfully and speedily adopted remote/digital methods to research the experiences of hospital doctors. In 2020, we used Zoom to conduct qualitative interviews with 48 hospital doctors; in 2021, we used Zoom and WhatsApp to conduct a Mobile Instant Messaging Ethnography with 28 hospital doctors. We explain how we adapted to a virtual setting and provide clear insights (case study vignettes) into the additional demands on researchers and respondents, in particular, the impact on the research team. Finally, we analyse the positive and negatives of using remote qualitative methods and highlight the potential of hybrid data collection models that combine remote and face-to-face methods. We also highlight our success in communicating findings to a policy audience, important in time-critical situations, such as the COVID-19 pandemic.

## Background

On the 12^th^ March 2020, to prevent the further spread of COVID-19, Ireland’s schools and workplaces were closed and stay-at-home orders issued for non-essential workers. Although initially intended as a short-term (17-day) measure, many of these restrictions remained in place for 22 months (in relation to working from home) and in relation to access to hospitals, remained in place at the time of writing (January 2022). From the outset, it was clear that the COVID-19 pandemic would pose a significant challenge to the Irish health system. Even pre-pandemic, Ireland’s hospitals were under considerable strain, operating at higher capacity and with fewer ICU beds than the EU average (Walsh et al., 2020). The risk that the pandemic would overwhelm the Irish hospital system was a key consideration in determining Ireland’s pandemic response.

As researchers focussed on understanding and depicting the working lives of Ireland’s hospital doctors, it was clear to the HDRM research team (HDRM is the Hospital Doctor Retention and Motivation research project; funded by the Irish Health Research Board and hosted by the RCSI Graduate School of Healthcare Management) that the pandemic would have a huge impact, both on their working lives and on the conduct of our research. Even before the pandemic, we found that hospital doctors frequently worked long hours with a high work intensity and that many struggled to achieve a work-life balance (Humphries et al., 2020). Although we were eager to ascertain the impact of the pandemic on the working lives of hospital doctors in order to inform medical workforce policy and practice, it was quickly apparent that undertaking qualitative face-to face research with doctors in their hospital workplaces would pose significant challenges and that a new approach was required. In this article we reflect on how our research practices changed, and how we and our respondents enabled such adaptations. We analyse the consequences and highlight how remote methods can be substituted with both costs and benefits for all.

## Revising the Approach; Using Remote Ethnography and Using Digital Methods

In our original design, we had adopted a range of observational and interview based methods to gain insights and a rich understanding of *‘work-as-done’* (Braithwaite & Rapport, 2021) by hospital doctors. This combined approach, usually labelled ethnography, typically involves researchers becoming *‘embedded in the workplace.* . . [to] *observe how work is enacted over time’* (Braithwaite & Rapport, 2021). Ethnographic methods have been used extensively and successfully in health (Cupit et al., 2018; Liberati et al., 2016; Liberati et al., 2019; Xyrichis et al., 2017) and health workforce (Becker et al., 1961; Darbyshire et al., 2020; Dixon-Woods et al., 2014; Jenkins, 2020) research worldwide. It soon became obvious that our fieldwork needed to be modified to comply with government COVID-19 guidelines on non-essential travel, working from home, hospital visitor restrictions and social distancing. The question for the research team was how we could become immersed in the daily working lives (O'Reilly, 2009) of hospital doctors, or understand what was happening (Cupit et al., 2018) in hospital settings during COVID-19, without being able to spend time *‘being there’* (Howlett, 2021). Could the research team build trust and rapport with respondents, gain insights into the workplace *without* meeting them face-to-face or visiting their workplaces?

In adapting the methods for a COVID-19 context, we drew early inspiration from Posthill’s work on remote ethnography. In his 2016 paper, he explained that remote ethnography involves using *‘whatever technical means will help us gain insights into the lives and deeds of our research respondents’* (Posthill, 2016). We subsequently reviewed (and continue to review) the emergent literature on conducting qualitative research during the COVID-19 pandemic (Ghosh, 2020; Howlett, 2021; Jowett, 2020; Kim et al., 2021; Lupton, 2020; Pocock et al., 2021; Rankl et al., 2021; Richardson et al., 2021; Vindrola-Padros et al., 2020) and a broader literature around the growing use and usefulness of digital and remote methods (Barratt, 2012; García et al., 2015; Gibson, 2020; Kaufmann & Peil, 2019).

Our pre-pandemic research plans had involved conducting short-term, focussed ethnographies (Knoblauch, 2005; Pink & Morgan, 2013) in three hospital settings. Following Pink et al., we considered short-term, multi-sited ethnography as a feasible way of *‘producing alternative ways of knowing’* (Pink & Morgan, 2013) about hospital doctors and their hospital work environments. Although initially disappointed to be unable to conduct an in-person hospital ethnography, we drew inspiration from digital ethnographers whose work demonstrated how ‘*learning ethnographically*’ (Varis, 2015) could be achieved remotely and who demonstrated that remote ethnography could facilitate access to otherwise inaccessible places or people (Plowman, 2017). We were also inspired by Vindrola-Padros and her team who conducted rapid, applied qualitative research in the early phase of the pandemic (Vindrola-Padros et al., 2020) and with other qualitative researchers who successfully adapted their methods to a pandemic context (Kim et al., 2021; Pocock et al., 2021). We were keen to conduct ethnography *with* rather than simply about respondents (Pink & Morgan, 2013), and to create a non-intrusive approach to data collection that ensured privacy, maximum flexibility and that was non-burdensome for respondents.

The literature also demonstrated that digital technologies, such as WhatsApp and online chat, could enable the generation of qualitative data in minimally invasive and collaborative ways (Barratt, 2012; Gibson, 2020) which are particularly suited for research with those who are comfortable/familiar with the technology (Gibson, 2020). For example WhatsApp has been praised for its flexibility and convenience as a research tool and is considered particularly useful for conducting research with time-pressured respondents (Kaufmann & Peil, 2019). In our previous face-to-face interviews we and others had noted that Irish hospital doctors used WhatsApp extensively at work as a means of workplace communication and information sharing (O’Sullivan et al., 2017). Thus, the fit for this set of research respondents seemed especially apt and the HDRM research team were confident that using remote ethnography would enable us to open *‘windows behind closed doors’* and to access a world (the hospital workplace) otherwise inaccessible (Meskell et al., 2021).

Our prior experience of conducting survey-based research with hospital doctors saw us receive articulate and thoughtful written insights in response to ‘free-text’ questions. Several earlier papers have drawn from these ‘free-text’ responses (Creese, Byrne, Matthews, et al., 2021; Humphries et al., 2020; Humphries et al., 2015). Our assumption was that hospital doctor respondents would be likely to provide in-depth, detailed written responses whether triggered by survey or online methods. We were confident that hospital doctors would also have the necessary means (mobile phones) and familiarity with the software (WhatsApp, Zoom) which are mainstream and have been in everyday use throughout the COVID-19 pandemic (Howlett, 2021).

Our team also had to reconsider access and recruitment. In March 2020, we were in the process of negotiating access to three hospital sites with the hope of collecting data during the summer of 2020. All recruitment strategies were designed pre-pandemic and on the presumption of the research team *‘being there’* (Howlett, 2021), on-site in hospitals to collect data. In order to collect data remotely (necessitated by the pandemic context), the research team relied heavily on a network of professional contacts, as highlighted by Howlett (Howlett, 2021). Despite the lead researcher having 15 years’ experience of health workforce research, strong links with postgraduate training bodies, a publication track record, and familiarity with recruiting research respondents in the past (using social media, e.g. Facebook, Twitter) (Humphries et al., 2015; Humphries et al., 2019; McAleese et al., 2016)) recruitment for the remote ethnography still took significantly longer than in non-pandemic times. This has implications for future study timelines and for those who are new to their fields of study, those have not yet developed contacts in the field and/or who have short-term research funding. Funders also need to be alerted to this reality.

Overall, the purpose of this article is four-fold. Firstly, to reveal key learning points through detailing how we successfully and speedily adapted our qualitative research methods to the challenging circumstances of the COVID-19 pandemic, using remote data collection methods during the COVID-19 pandemic. This is an account of rescuing and modifying a research study mid-implementation. Here we are adding to the evidence base about when/whether to use remote qualitative methods. Secondly, we want to add to the evidence base about how to make remote qualitative methods work and to identify key process issues that need to be planned and managed when using remote methods: the need to match respondent familiarity and capability (especially in relation to digital technology) to the selected approach; the issue of recruiting respondents and privacy and ethical issues. Thirdly we provide clear insights through our case study vignettes of the demands put upon both researchers and those being researched by using such digital tools and remote practices: issues of unsocial hours of work and the impact of flexible working; intrusion of work into the lives of researchers; and the necessity of supporting and debriefing of remote researchers in handling difficult or emotionally charged conversations. Whilst these challenges are not unique to using remote methods, they can, nevertheless, be accentuated and overlooked in the implementation of such methods. The issue of power relationships and dynamics between researcher and the researched also arise and crucially can affect the wellbeing of all. Lastly, we briefly analyse some of the positive and negative realities of using remote research practices with professional respondents and query whether future, intentional designs might incorporate hybrid models of data collection, which combines some of the assets of remote and of face-to-face data collection methods.

## Methods

**Figure 1.**
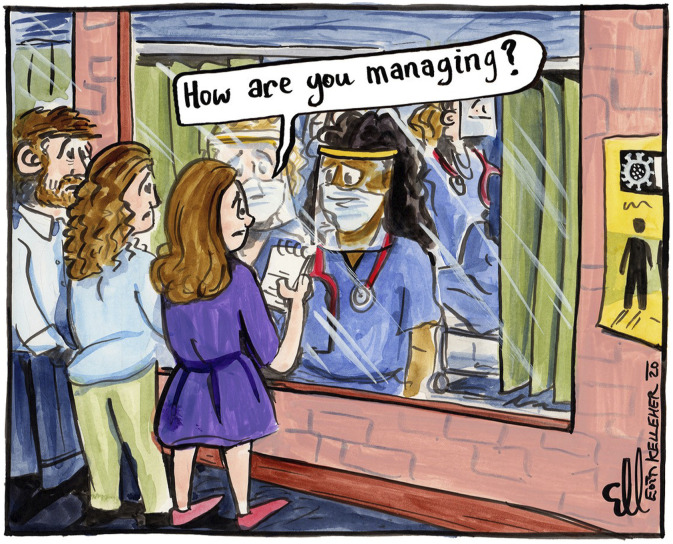
Cartoon (by @EoinKr) depicting the COVID-Hospital doctor retention and motivation Research Process.

**Figure 2.**
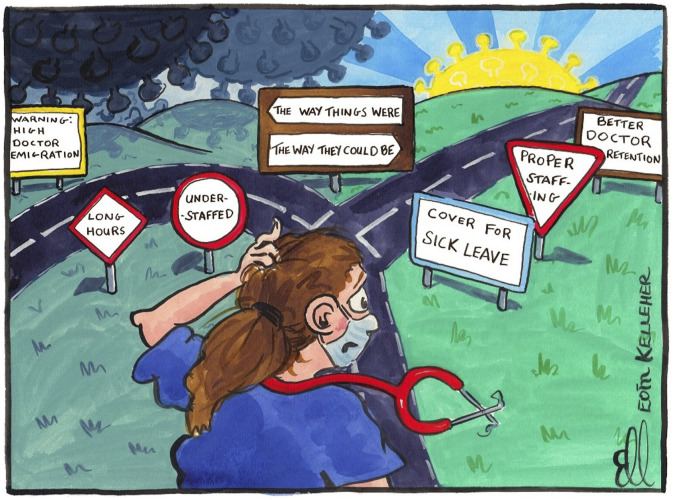
Cartoon (by @EoinKr) depicting initial research findings from COVID-Hospital doctor retention and motivation.

In terms of remote methods that would enable us to overcome the practical challenges of data collection during a pandemic, we opted for Zoom interviews with hospital doctors (Project 1) and a Mobile Instant Messaging Ethnography (MIME) using Zoom and WhatsApp (Project 2). Insofar as it was possible, we sought to stagger data collection to avoid the COVID-19 pandemic ‘peaks’; Project 1 ran from June to July 2020 (between wave 1 and 2) and involved Zoom interviews with 48 hospital doctors. Project 2 ran from June to December 2021 (following a successful national vaccination campaign) and involved a remote ethnography with 28 hospital doctors. Profiles of the respondents recruited for projects 1 and 2, are contained in Tables 1 and 2. Building on the work of Kaufmann et al. (Kaufmann & Peil, 2019), the HDRM team used WhatsApp to undertake a 12-week MIME. Both projects received institutional ethics approval from the Royal College of Physicians of Ireland Research Ethics Committee; project 1 in May 2020 and project 2 in June 2021. Each project used social medial (Twitter) to recruit hospital doctor respondents, using cartoons (Figures 1 and 2) and opinion pieces (Humphries, 2021; Humphries, Creese, Byrne, & Costello, 2021) to bolster recruitment. The recruitment process for project 2 was more difficult than for project 1; it took 3 months to recruit 28 hospital doctors. We believe that timing was likely a contributory factor as data collection coincided with pandemic fatigue within the medical workforce, a cyber-attack on the national health service (May 2021) which caused most of its IT systems to be shut down, summer-time annual leave and the annual changeover of junior hospital doctors (in mid-July). In relation to respondent recruitment, in line with Richardson et al., we found that recruitment was greatly facilitated by relationships built before the COVID-19 pandemic (Richardson et al., 2021). As we recruited, we were keenly aware that our respondents were all front-line hospital doctors working through the COVID-19 pandemic and those recruited are those who stepped up and kindly agreed to participate despite the competing (and urgent) demands on their time. In both study 1 and study 2, we interviewed more female than male doctors (See Tables 1 and 2). Although internationally trained doctors comprise 43% of the Irish medical workforce (Medical Council of Ireland, 2020), we failed to recruit internationally trained doctors to participate in the study. In terms of seniority, we managed to interview a balance of junior hospital doctors and consultants in study 2, with more junior hospital doctors than consultants participating in study 1 (see Tables 1 and 2). In our original study design for study 2 we had planned to recruit 10 hospital doctors at one rural hospital site; in our remote ethnography, we recruited 9 respondents from rural hospital sites and 19 from urban hospital sites.

**Table 1.** Respondent Table HDRM Study 1 (*N* = 48).

Respondent table HDRM study 1 (*N* = 48)			
Gender	Male Female	11 37	48
Caring responsibilities	Yes No	15 23	48
Current grade	Consultant (senior doctor) NCHD (junior doctor)	17 31	48
Country of training	Ireland Outside Ireland	47 1	48

**Table 2.** Respondent Table HDRM MIME/Study 2 (*N* = 28).

Respondent table HDRM study 2 (*N* = 28)			
Gender	Male Female	8 20	28
Caring responsibilities	Yes No	10 18	28
Current grade	Consultant (senior doctor) NCHD (junior doctor) Associate specialist (staff grade)	13 14 1	28
Location	Urban hospital Rural hospital	19 9	28
Country of training	Ireland	28	28
Data collection log	Interview 1 WhatsApp/MIME* Interview 2	28 24 24

Data collection were conducted by three HDRM researchers (NH, JPB, JC) whilst working from home in line with government COVID-19 restrictions 2020-21. In terms of the professional backgrounds of the research team, the team comprised of a sociologist with health workforce expertise, a social anthropologist and a sociologist. Although none of the team were clinicians, all were working on a long-term research project focussed on hospital doctors and so had some level of familiarity with the hospital workplace contexts and with the work-related challenges faced by respondents. All qualitative interviews were conducted via Zoom/by phone and guided by theme sheets which focussed primarily on respondent’s experience of working as a doctor during the COVID-19 pandemic. Prior to interview, each respondent received an information leaflet and completed an online consent form. Interviews were audio recorded and transcribed by an external company. The use of secure servers, end-to-encryption and a confidentiality agreement ensured the confidentiality of data during the transcription process. Transcription was undertaken in parallel to interviews, which was an efficient use of time. Transcripts were then de-identified by the research team and offered to respondents for approval/amendment. The research team used MaxQDA software to support data analysis, using open coding (Strauss & Corbin, 1990) and inductive analysis (Glaser & Strauss, 1967) for the dataset before using thematic analysis (Braun & Clarke, 2006).

**Figure 3.**
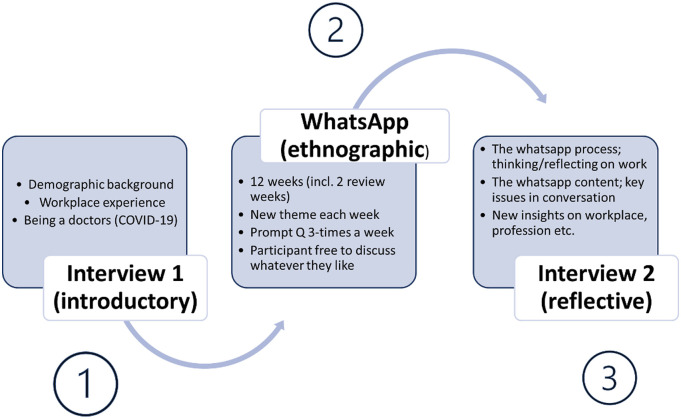
Hospital doctor retention and motivation mobile instant messaging ethnography (project 2) study process.

Project 2 involved 28 respondents, each of whom were invited to take part in two interviews and a 12-week MIME (see Figure 3). Mobile instant messaging ethnography was a WhatsApp conversation between respondent and researcher, guided by a theme sheet which involved researchers sending 3 messages per week to respondents, with a distinct theme covered each week (workplace interactions, work-life balance, etc.). For project 2, each researcher was issued a separate phone number and used a separate mobile phone for the purpose of data collection. These phones were screen-locked when not in use, requiring a 4-digit pin and/or fingerprint scan to unlock and were stored securely in researchers’ homes for the duration of project 2. Respondent hospital doctors were encouraged to reply to WhatsApp messages from the research team whenever they had the time/space to do so. Replies usually triggered a short WhatsApp conversation between respondent and researcher. Respondents were also interviewed before and after their participation in MIME, with the final interviews providing an opportunity to reflect and discuss some of the issues raised via WhatsApp.

To disseminate the findings of project 1, three separate writing teams were established to enable analysis and write-up of three papers to occur simultaneously. Timing was important to ensure that findings were available to inform the ongoing pandemic response. The establishment of writing teams also ensured that co-authors (academics and clinicians with other work commitments) did not become over-burdened. Three open access articles were published between February and July 2021 (Byrne et al., 2021); Creese, Byrne, Conway, et al., 2021; Humphries, Creese, Byrne, & Connell, 2021, along with three research briefs (see https://www.rcpi.ie/hdrm/) which were circulated and discussed with key policy makers. Two opinion pieces were published in early 2021 to further raise the profile of the research project and disseminate findings (Humphries, 2021; Humphries et al., 2021). At the time of writing, project 2 data collection is ongoing, but we envisage a similar process of analysis, write-up and dissemination. In terms of this paper, data collection was conducted by NH, JPB and JC; LMcK worked with the team to shape the paper, bringing valuable ethnographic expertise to bear on the analysis and write-up process.

### Findings

Here, we reflect on the key learning points from our adaptation to using remote qualitative research methods and outline what worked and what did not. Although data collection in project 2 recently concluded (end December 2021), we explore what lessons have emerged about the adaptation process, including: the decisions that arose about new practices; the challenges of remote recruitment; ethics: privacy; and the preparation, skills and resources needed within the research team to successfully adapt to a remote way of qualitative researching. We firstly include some reflective vignettes from each member of the research team on their experiences of using remote ethnography (project 2/MIME method). Individual researchers are not identified in order to protect respondent identities. These case examples highlight many sensitive and raw aspects of remote data collection during the COVID-19 pandemic including that of researcher and researched vulnerability; the openness, deep emotions and fulsome disclosure that can be achieved via digital communication; the demands for researchers to show empathy and listening/counselling skills; the immediacy and realities of workplace stress; and how the researchers themselves can feel powerless without familiar face-to-face cues during the interview process that allow them to moderate their questions and reactions.

## Researcher Reflections and Experiences of Remote Ethnography

As soon as the MIME WhatsApp message exchanges began, it was clear to the research team that MIME was a successful means of connecting and communicating with hospital doctors during the COVID-19 pandemic. It was also clear that respondents would share their work-related distress and their frustrations of working within the Irish health system with us and that we needed to find a way to process that information and our own emotional responses to it. This need was identified by the newest member of the team (JC), drawing on her feminist approach to anthropology. It was also informed by her reaction (as a migrant recently arrived from Australia) to the workplace experiences of hospital doctors within the Irish health system, challenges that the other team members were, unfortunately, more familiar with. We established a weekly research team ‘debrief’ meeting to support us through the data collection process during projects 1 and 2. These team meetings provided team members with some time and space to (begin to) process our emotional responses to data collection. We met weekly, often outdoors (in line with COVID-19 restrictions) and talked through our experiences of data collection; the interactions that had stayed with us. These meetings became an important space to share stories, provide peer support and to acknowledge some of the *‘the difficult emotional encounters we face in our ethnographic and indeed wider qualitative work’* (McMurray, 2021). Within these meetings, team members encouraged each other to further develop and write-up reflections discussed within the group. Below are three vignettes, each selected by a member of the team to capture their early emotional responses to the content and the process of MIME data collection. They highlight the challenges we faced as a team and conclude by outlining some of the learning points we have taken from that process.

### 1. Uncovering Deep Negative Emotions: the Researcher as Counsellor

We began our WhatsApp ethnography with some general, ease-in questions. Innocently one afternoon, I ask each respondent:

*Researcher: ‘What was the most uplifting aspect of your workday today?’.*

*R17: ‘Nothing. Today was a very bad day…’*

*R17: Details a major personal issue, then adds:*

*R17: ‘Today was one of the worst days of my life and honestly even though the workday went well and I completed six difficult procedures successfully I cannot say anything can uplift me because my mood inside was distraught.’*

Instantly my stomach drops – the respondent has shared this grief with me, but what am I expected, or able, to do in return? Tentatively, I message back,

*Researcher: ‘I am so sorry. That's so hard. Is there someone you can talk to about it?’*

*R17: ‘No. I told nobody.’*

And it hits me, hard, that I am the only person this respondent has shared their grief with; yet, I am an ethnographer, not a counsellor, and not equipped at all to help them deal with this grief constructively, or to ensure they really are okay. That is immediately an anxiety-provoking thought.

It is perhaps an appealing thought - sharing grief through an anonymous, secure social media channel with someone who is ethically bound not to tell anyone else, who has no stake in the issue on either a professional or personal level. One respondent who faced challenging and upsetting situations at work said,

*R13: ‘I try not to bring up work issues with family… [and] HR and other non-clinical managers are rarely helpful’.*

Another suggested they could not share feelings with colleagues, still less with their organisationally provided counselling services, because they feared that their employer would use it against them:

*R17: ‘I don't trust them, they use their counselling service as an excuse to get rid of "troublesome" staff’.*

Certainly face-to-face, as an ethnographer and interviewer I have listened to research respondents’ stories before, I have passed tissues, provided chocolate, and otherwise helped with the processing of difficult stories and experiences together. But unlike a counsellor, my role is to take stories, experiences and explanations and draw out common themes and threads across multiple respondents. In face-to-face processes I could support research respondents by listening. Using remote methods is a sometimes disjointed, and often asynchronous, process in which it is impossible to effectively support respondents and equally impossible to see how a respondent is coping. One WhatsApp respondent sent a flurry of late-night messages:

*R11:’It’s been a really rough year’*

*R:11 ‘we’re all burned out… I’ve gone from someone who was happily jumping out of bed to work to dreading it’.*

Tentatively I asked if they were upset and whether they had someone to talk to. I spent an anxious night worrying before getting a response the following morning:

*R11: ‘Not upset, it’s something I’ve very much come to terms with.’*

Somewhat of a relief – but without the tools or the mandate to make sure respondents really are okay, the ethnographer is left wondering whether they are truly ethically caring for their respondents, or indeed, themselves.

### 2. The Hazards of Instant Connectivity: The Angry (and Guilty) Researcher

I had not anticipated some of the ways in which the MIME method would differ from ‘live’ ethnographic or qualitative research and how much it would affect me. I had simply hoped that it would be a new and interesting way of generating data during the COVID-19 pandemic. An advantage of MIME which became immediately apparent, was the feeling of connecting with respondents ‘live’ (or almost live). Once they had agreed to take part, respondents freely shared insights from their working day, frequently bowling us over with their candid responses. Unlike interviews, where respondents might reflect on events or experiences from weeks or even months ago, MIME allowed respondents to tell the researcher how they feel about work right now/in the moment and to continue to do this over a 12-week period. This enabled the research team to share with them the highs and lows of their working lives throughout that time.

But at times, this feeling of connection, a distinct advantage of MIME, felt like a disadvantage. It sometimes felt that respondents used MIME to share work experiences with the research team that they may not have fully processed themselves. This can occasionally lend a ‘rawness’ to the MIME conversations and a sense of hearing directly from workers/a workforce-in-pain. For instance,

*R5: ‘today was probably one of the most challenging workdays I’ve ever had . . .’*

*R16: ‘I worked 10hrs on the floor and still huge waits to be seen. Left destroyed and exhausted’.*

On occasion, I have found it hard to respond to messages like these. I find myself typing, deleting and re-typing responses and hoping that they are appropriate. I realise that I am searching for responses that will do no (further) harm. I find myself explaining to respondents that we aim to give voice to their experiences, that we will share them with policy makers. I am not sure whether I am trying to convince them (or convince myself) that their stories and our research will prompt policy makers to improve the working conditions of Ireland’s hospital doctors.

The challenge for me has been how to process my own frustration at their experiences of the workplace, knowing that employees in other sectors (including my own) expect and receive better treatment in the workplace than is standard for hospital doctors. I am angry that Ireland has a health system that does not adequately support its hospital doctors. I am frustrated for their patients and for Irish society more widely who are often unaware both of how bad things are within the health system and how much better they could be. I want to use this research to somehow ‘fix’ the challenges within the health system and I feel guilty at the prospect of not being able to achieve this. I am also angry that many of the challenges raised by respondents in their MIME messages relate to long-standing problems in the health system which are widely known, rarely acknowledged and which remain stubbornly unresolved.

### 3. Waiting and Worrying for Vicarious Interactions: The Empathetic Researcher

On a Monday evening in July, I sent out my first round of questions to doctors via WhatsApp. What followed was an unexpectedly anxious wait, checking those blue ticks (signalling the message has been read), waiting for a response. I was worried; What if they did not respond at all? What if all I got back was one word? Thankfully, these concerns did not materialise as each respondent responded and each conversation developed its own rhythm. At times I have not received any responses for hours or even days. My task then became waiting – an unusual role for a researcher whilst in data collection mode. Check those ticks again; wait for that phone notification sound. Conversely, there have been occasions when I have received several detailed responses from doctors within the same hour or two. These responses usually require follow-up questions or clarifications. These simultaneous conversations then dominate my time and attention as each requires a tailored response. I have found myself responding to texts just before midnight in bed, and regularly for short periods of time at the weekends. In most cases this attempt to ‘catch’ respondents when they reply has led to longer conversations and the generation of further data, but this ‘feast or famine’ type data collection requires a large deal of flexibility on the part of the researcher.

Unlike face-to-face interviews, or observations, MIME relies heavily on text. When reading respondent responses, it can be quite difficult to know the intonation or tone of statements – especially when negative. I have found it very difficult to balance the need for support or empathy, with the opportunity to probe further into the issues identified. This is a very tricky process, shaped by a limited knowledge of the individual respondents and their communication style. For example,

*Researcher: ‘How did you feel at work today?’*

*R26: ‘Terrible, guilt ridden and under pressure. . . I felt angry and resentful . . . my empathy and compassion have worn away’*

*Discussing their experience of working through the third COVID-19 lockdown in Ireland (Winter 2020), another respondent noted:*

*R7: ‘. . . we did not sign up to become punching bags for the failings of the Health Service’*

In both cases, respondents went on to explain these answers, but I still found myself staring at the phone, wondering what the most appropriate response should be. It felt exploitative to ask these doctors to dwell further on negative experiences. Were my questions helping, or adding to their misery? Striking that balance is made more difficult without a shared physical space and presence.

The smartphone represents a constant contact point with respondents. WhatsApp allows me to scroll through what was said, whenever I want. This has meant that the MIME data have had a more enduring effect on me. For example one doctor who had initially responded promptly went very quiet after describing the busyness of work and how overwhelming it could be. Two weeks and five unanswered questions later, I was beginning to worry whether they were okay and getting the support they needed. Suddenly, late one Tuesday evening, I received very detailed and considered answers to all questions. Although this was a relief, their responses reflected on whether the sacrifices made for work were worthwhile. In some ways I feel like I am on a reflective journey with this doctor.

## Learning from Reflexivity

As a team, our weekly debriefs enabled us to reflect on the research process; to build reflexivity into our practice and provided the time and space to recognise and reflect upon the *‘emotional impact of our research’* (McMurray, 2021). We would encourage research teams to conduct regular debriefs and to ensure that formal/informal emotional supports are made available to researchers and data collectors to help to protect their emotional (as well as physical) wellbeing throughout the research process. The debriefing process helped the team to identify the issues arising from the content or the process of the research that were causing us concern. As highlighted in the second vignette (see above), research impact arose as a concern. As a result, we feel that it is important to be realistic with the research team (and with ourselves) about the capacity of any research project to inform real world change in a short time-frame. Despite our ambitious plans for knowledge exchange and the fact that we are in regular communication with policy makers in the *hope* of informing change, we cannot guarantee this outcome. So although we are hearing a level of work-related (di)stress from our respondents, we cannot guarantee that our research will inform the necessary improvements in their working conditions. Our advice to future researchers is to strive to inform policy change and health system improvement, but perhaps to measure research impact by the extent to which you have communicated your findings to diverse audiences and to policymakers, rather than the achievement of policy improvement (which are beyond our control). A related issue for the research team was knowing how to respond to messages of distress from respondents. We found that conducting qualitative interviews with respondents before beginning MIME helped us to get to know respondents, which (somewhat) helped us to contextualise the messages. As a team, we also discussed how best to respond to different ‘types’ of MIME messages and in that way learnt from each other. Perhaps more formalised guidance, containing some sample messages/responses, developed as projects evolve, would be a useful support for other teams using MIME. The final interviews with respondents enabled the research team to re-connect with respondents to reflect further on the issues that had arisen in MIME. A logistical issue that also arose for the research team was that convenience and flexibility of MIME from a respondent perspective, can pose a challenge for the research team who may receive messages outside their regular working hours. Researchers may want to reply to respondents in a timely manner, but also need to maintain their work/non-work boundaries. Our solution was to operate a ‘time off in lieu’ for the research team throughout the data collection phase, so that researchers could reclaim non-work time.

## Remote Methods: What Worked Well and What Proved Difficult

### Speed and Implementation of Methods

For the HDRM team, the overwhelming advantage of adopting remote research methods was that it enabled us to sustain the project and quickly generate data during the COVID-19 pandemic whilst enabling both respondents and researchers to remain compliant with all relevant COVID-19 protocols (such as working from home, restricted access to hospitals and a ban on non-essential travel). As Pocock et al. explained, the pandemic gave qualitative researchers a reason to try virtual qualitative methods (Pocock et al., 2021). Remote methods enabled HDRM to generate rich, timely insights and *‘organisational intelligence’* (Dixon-Woods et al., 2014) on the challenges faced by the hospital medical workforce during the COVID-19 pandemic. This account of adaptive change in research design builds on the need for immediacy in reporting real life experiences in fast changing situations and on the imperative to produce timely insights for a wide audience including medical professionals, medical policy makers and health care managers. Another advantage of remote methods is that, as we have demonstrated, they can be rolled out quite quickly, particularly during the pandemic which enhanced digital literacy and increased familiarity with the technology used (Zoom, WhatsApp). Importantly, our direct experiences of remote methods have revealed how remote methods could offer researchers alternatives to (or a means of enhancing) face-to-face research and has revealed some practical ways of gathering first-hand data in emergency contexts.

### Keeping the ‘Show on the Road’

Critically, the roll-out of remote methods enabled the HDRM research team to function and to continue to deliver the project during a global pandemic, enabling us to keep to funding schedules, retain engagement in the project and maintain all team members in employment whilst working from home. Interestingly, the use of remote methods (Zoom interviews and WhatsApp messaging) could be used flexibly to accommodate both the changes to respondents’ and researchers’ schedules. For example two team members (NH, JC) needed to adjust their working hours around childcare/home schooling due to school closures (Ireland’s schools were closed for 8 months in 2020-21 due to COVID-19). For the duration of Project 1, HDRM regular team meetings shifted online (via Teams or Zoom platforms) where we shared research findings and data analysis, and planned research outputs and dissemination activities. Since May 2021, as guidelines permitted, we opted for in-person team meetings wherever possible (initially outdoors, moving indoors from September 2021 in line with guidelines). The ability to discuss the research findings, research process and recruitment strategies in-person and as a team has been hugely beneficial, particularly as the data collection for project 1 and 2 were completed remotely and without regular access to shared office space.

### Accessing Busy (and Geographically Dispersed) Respondents

Our reflections suggest that MIME, as a remote method, has proved to be less intrusive than an ethnographic observation study would have been. This was clearly essential during the COVID-19 pandemic when hospitals were inaccessible to non-essential staff/visitors for reasons of infection control and when staff were experiencing intensive workloads. This may apply in other hazardous situations where the presence of a researcher may prove problematic. The transfer to remote methods and digital tools in MIME facilitated a long-term but less time-intensive interaction with respondents. Despite the research team not being able to observe or shadow hospital doctors in their hospital workplaces, they interacted with respondents over an extended 12-week period. This helped the HDRM team to build rapport with respondents that they had never met face-to-face. Jaymelee et al. describe these virtual interactions as *‘ethnographic relationships confined to digital places’* (Kim et al., 2021).The longitudinal component of MIME complements and enhanced the insights gained from the initial qualitative in-depth interviews with respondents. Although we were not physically present alongside the doctors in their hospital workplaces, we spent a long time communicating with them about work via WhatsApp and hearing their response to issues as they arose (e.g. the cyber-attack on the HSE, the Sláintecare consultant contracts). Despite losing the comparative 3 hospital approach originally planned, the geographic reach of the project was also extended by use of digital technology. Whereas a traditional face-to-face ethnography would have been restricted to three hospital sites, perhaps one in a rural setting, the use of remote methods meant that doctors from rural and non-teaching as well as both junior doctors and consultants could participate with equal ease (see Tables 1 and 2). Mobile instant messaging ethnography could be considered an institutional ethnography albeit with the Health Service Executive (the national health employer in Ireland) rather than individual hospital sites, as the institution.

### Data Quality

In terms of the content and quality of data generated by remote methods, we judge that the adaptations have been a resounding success for the HDRM project (Project 1 and Project 2). In Zoom interviews and in their MIME/WhatsApp messages, respondents have given us incredible and immediate insights into their working lives. For instance,

*‘Researcher: Do staffing levels have a significant impact on your experience of work? How?*

*R9: Staffing levels have a massive impact on my experience at work. Thinking back to the times that I have felt most stressed, most under pressure and most frustrated at work – these times are all the times when there is more work than I can do safely myself. The list of jobs to do can become paralysing – there can be too many jobs to safely prioritise, and some of the frustration is because I know I wasn’t providing safe, optimum care’*

As we had hoped and anticipated, respondents were familiar with and comfortable using digital technology (Zoom, WhatsApp) and quickly embraced remote methods, sharing photos, voice notes and images with us as well as text. We did not anticipate how quickly respondents would openly share their reflexive insights into their working lives, seemingly getting straight to the heart of things. For instance,

*R16: ‘Today is better. We’ve been very overstretched this week with numbers incoming and I’ve taken that stress home. And I feel guilty about that. But I’m trying to be a bit compassionate towards myself. It’s impossible not to absorb all the misery of unmet need and not bring it home’.*

*R26: ‘I know you can’t change the system in the short term, it feels at least like someone is listening’.*

We have been taken aback at the trust that respondents have placed in us and the efforts they have gone to describe their work and their feelings about work. We are, however, reluctant to chalk this up as a success, as it has been truly awful to hear the struggles of frontline health workers. However, we feel that the connection between researchers and respondents has been helped by the relatively informal nature of our WhatsApp conversations; the immediacy of the MIME method and the fact that researchers provided an outlet and perhaps a release for hospital doctors who have spent the past 2 years working through a global pandemic, and finally the fact that respondents value our research enough to participate.

### Managing Researcher workloads

In terms of managing researcher workloads, there were pros and cons to the use of remote methods. With face-to-face ethnography, researchers and respondents typically negotiate the timing of fieldwork or data collection. Researchers tend to ‘block’ time for fieldwork, in the knowledge that fieldwork is an all-consuming, intensive activity. Whether living at the research site or visiting it regularly to collect data; researchers tend to prioritise or focus exclusively on the fieldwork. This exclusive focus was difficult/impossible to achieve during the MIME study. The combination of the need to work from home during the pandemic and the use of digital technology for data collection meant that the boundaries between work and non-work time were blurred for the research team.

The advantage of this was that data collection could take place in parallel to domestic responsibilities (particularly the unanticipated additional childcare and home-schooling responsibilities during the COVID-19); the disadvantage was that in terms of scheduling, the researcher *only* had control over when messages were sent via WhatsApp and no control over when respondents would reply. Sometimes respondents replied promptly, sometimes a few hours or days later. To maintain the conversational tone of MIME and to build and maintain rapport with respondents, HDRM researchers developed a policy of endeavouring to reply and/or acknowledge any WhatsApp reply sent by respondents shortly after messages were received. This proved especially difficult when researching a group (such as hospital doctors) who work long and unpredictable hours and/or in shift patterns. By way of illustration, in 1 week of data collection (the first week September 2021) 20/28 respondents sent replies outside normal working hours (9–5). This meant that WhatsApp messages could arrive at unsocial hours (late night/early morning), potentially disrupting or intruding on researchers’ lives. Although we established an informal ‘time off in lieu’ system, which allowed team members to take time off work in return for non-work time spent replying to MIME messages, data collection was a priority with researchers continuing data collection during periods of annual leave and at weekends, so as not to interrupt the data collection schedules or inconvenience respondents. Whilst this demonstrates our dedication to this piece of research (and mirrors the tendency of many hospital doctors to work beyond scheduled working hours in the delivery of patient care), it also is something that future researchers should take note of and seek to counter-balance by actively monitoring working time and ensuring that the research team are each getting adequate rest time to avoid exhaustion and burnout.

A related issue was the logistical challenge of managing MIME WhatsApp conversations for several respondents at the same time, with each on slightly different schedules. To counter this and to prevent overload we set 10 as the maximum number of MIME respondents that any HDRM researcher could/should co-ordinate at one time. Even with these checks and balances, data collection was fairly intensive for the research team at various stages during project 2, confounded by the fact that we were never ‘only’ doing data collection, but also completing funding proposals, journal articles, and other work tasks. Because MIME data collection occurred on mobile phones and laptops, there was a tendency to reply to work-related emails and complete other work tasks alongside MIME. Again, this would not occur in a face-to-face ethnography where fieldwork would receive the researchers’ full and undivided attention. We would recommend that future researchers anticipate these logistical challenges and set out a research protocol to address them.

### Supporting Researcher Wellbeing

During project 1 and 2, the HDRM team organised regular team meetings and de-briefings to help us to process some of our frustration, sadness and concern at issues raised by respondents (see reflective vignettes above for more detail). We recognised the need not to ignore the emotional aspect of doing qualitative or ethnographic fieldwork (McMurray, 2021), but also the added burden(s) of conducting that fieldwork with health-workers during a global pandemic. The physical distance between researcher and respondent during data collection altered the dynamic in ways we did not anticipate and which should be factored into future MIME studies. When respondents communicated via WhatsApp, they messaged us directly from (or just having completed) their working day. This means that researchers received an immediate snapshot of how they felt at that moment without any processing (or sugar-coating) that might occur in the run up to an interview scheduled in advance. In this way MIME mirrors a face-to-face ethnography and additional supports are required to ensure the continued wellbeing of researchers during data collection. Although remote ethnography caries fewer risks to the *physical* safety of researchers, the emotional risks do not disappear and may even be intensified in a remote setting where researchers are working from home without immediate access to a shared presence and/or physical cues as well as the informal support of colleagues. Another aspect of MIME, highlighted in the vignettes, is that the discussion between researcher and respondent is immediately available for review and analysis (within the WhatsApp chat), which can make it easier for researchers to revisit research interactions which might have been fleeting in a face-to-face context. Again, peer support was vital to the HDRM team in helping us to navigate the unfamiliarity of a new method (MIME) in a new context (during a pandemic whilst working from home) and in helping us to process our emotional responses to the data collected. Future researchers might formalise this support in advance and ensure that all members of the research team feel adequately supported throughout the data collection process.

### Data Privacy and Ethical Issues

Ethics approval for project 1 and project 2 were received from the Royal College of Physicians of Ireland research ethics committee. In terms of privacy, an advantage of remote ethnography is that respondents did not have to publicly acknowledge their participation in the HDRM study, as they would if they were being shadowed at work by a HDRM researcher. We imagine that this made participation a more attractive option for some respondents. The research team were working from home for the duration of project 1 and project 2. All data were stored on work-purchased encrypted laptops. With privacy concerns in mind, and to facilitate the division of work and non-work for the research team, each member of the team used a separate mobile phone and phone number for the purposes of data collection. This meant that the research team did not need to share their personal telephone number with respondents and that they had a ‘work phone’ for the purpose of data collection to attempt to prevent data collection from inadvertently intruding on personal time (albeit with limited success, as evident from earlier discussion). Each member of the team also had WhatsApp installed on their laptop, so that they could use either phone or laptop to communicate with respondents. On a regular basis, a transcript of the WhatsApp conversation was downloaded from the mobile phone and saved on a shared HDRM team folder. Interview transcripts were also stored in this folder. Once the MIME process was complete, each researcher de-identified both interview and WhatsApp transcripts, removing reference to individuals or individual hospital sites (or anything identifiable), transcripts were then shared with respondents for approval.

### Remote Communication and Rapport

Despite the high quality of the reflections and data generated, building rapport between researcher and respondent via remote methods was a mixed experience. In particular, the research team found that conducting qualitative interviews via Zoom was not quite as good as conducting them face-to-face and that the online setting occasionally dulled the interaction, despite our familiarity with remote communication during the COVID-19 pandemic. This is supported by Meskell et al. who explain that *‘in an online environment our honed skills of communication and empathy are dulled, we are unable to fully determine the atmosphere in the room’* (Meskell et al., 2021). Interestingly the HDRM team found that rapport was *more easily* developed with respondents via WhatsApp. We suspect that this may relate to the informality or familiarity of WhatsApp as a widely used means of daily communication, embedded in our everyday life – our WhatsApp conversations often felt like a work-related ‘chat’ between researcher and respondent. WhatsApp seemed to suit respondents as well, with several remarking on the flexibility of being able to interact with the research team simply by sending a WhatsApp. This meant that they could reply to our messages as soon as something relevant struck them, for instance one respondent sent a photo of a hospital filing cabinet with no drawers allocated to doctors. This photograph was unsolicited but clearly illustrated the respondents point – that the hospital workspace was designed without doctors in mind. This seemed to enhance the high level of ‘buy-in’ and commitment to the research project and a willingness to embrace its’ innovative methods.

### Researcher and Researched; Balancing Schedules, Workloads and Agency

It has also been noted that remote methods can enhance the agency of respondents over the research process, giving them more control over when (or if) to reply to messages or how fully to participate (Howlett, 2021). Much has been written about the distribution of power and agency between researcher and researched in terms of face-to-face qualitative methods (see (Bell & Roberts, 1984; Oakley, 1981, 2016)) with the researcher having control over the interview process. We suggest that there may be a levelling effect with remote methods, particularly MIME. In developing MIME, we sought to develop a method that was ‘respondent-centred’ and as flexible as possible, an important consideration in encouraging participation from respondents who are already busy balancing multiple, competing priorities, as Kaufmann and Peil (2019) noted. However, as we noted above, MIME brought a mix of costs and benefits for the researchers in terms of their own work scheduling and work-life balance, which we raise again below. Our findings reinforce other studies which have shown that using digital technology for data collection (such as WhatsApp, Zoom, Twitter) works especially well with respondents with a high level of familiarity, literacy, access and competency with its usage (Gibson, 2020; O’Sullivan et al., 2017) and this was another aspect to our ‘respondent centred approach’.

### Recruitment and Retention Challenges

Despite the flexibility of remote and digital methods, the team struggled to recruit respondents, particularly for project 2. Even as a high profile research team with strong contacts in the field, it took us 3 months (June, July, August 2021) to recruit 28 hospital doctors. We are uncertain whether these recruitment difficulties related to the timing of the project; the wider pandemic context and/or a reluctance among respondents to commit to 12 weeks of data collection. Within the 28 doctors recruited, we recruited both consultants and junior hospital doctors and sought to achieve a gender balance. However, despite our contacts in the medical profession and our experience of recruiting hospital doctors for similar studies, we struggled to recruit a sample that fully reflects the medical workforce in Ireland’s hospitals. For instance, we recruited no non-EU hospital doctors to Project 1 or Project 2, despite the fact that non-EU hospital doctors make up 43% of the medical workforce in Ireland (Medical Council of Ireland, 2020). Although recruitment challenges were undoubtedly complicated during the pandemic, recruitment challenges should also be considered by future researchers considering the use of remote recruitment as it might be even more difficult for research teams without pre-existing contacts.

Retaining respondents in the study has also been a challenge which relates to the ongoing pandemic and the busy working lives of respondents. Over the course of the MIME study, several respondents lost contact with the research team for a week or two, usually citing heavy workload pressures. The HDRM team offered flexibility to respondents, offering to pause participation in MIME whenever needed and enabling them to re-start their participation once workload pressures had eased. In the end, two respondents formally withdrawn from the study, both citing burnout as the reason, whilst another two respondents dropped out of the study. Each of these four respondents had completed one interview and several weeks of MIME before withdrawing. In terms of our duty of care to research respondents, it is important for future researchers to be aware of the emotional impact that participation may have for respondents and to have protocols in place to mitigate them and also, as in all research projects, to remain open to respondents pausing or ceasing participation in the study at any time.

### Losing the Workplace Context

The COVID-19 pandemic restricted organisational access for the HDRM team and whilst MIME enabled us to substitute organisational access for individual access (and observational data for reflective data), it did mean that the organisational and workplace context is hard to interpret. For all the benefits of MIME and remote methods, they did not enable the HDRM team to undertake a hospital level workplace ethnography, as had originally been planned for project 2. Adopting remote methods forced us to sacrifice descriptive/observational insights into hospital level workplaces and the ability to draw comparisons between hospital workplaces or hospital cultures. Although MIME enabled us to relate to the experience of the *individual* doctor within a given hospital, these accounts are somewhat de-contextualised as each doctor is situated within a different hospital/unit workplace. Perhaps a hospital-specific version of MIME could have generated more workplace level or organisational insights. Due to the ongoing pandemic, the team opted not to restrict recruitment to a specific hospital or specific speciality in anticipation of recruitment challenges in the context of the COVID-19 pandemic (see above). Recruiting from three hospital sites (or from one speciality) would have created extra workload for staff charged with gatekeeping for the project at a time when they (and their hospitals) were singularly focussed on managing the challenges posed by COVID-19. In a non-COVID context perhaps MIME could be used alongside a face-to-face hospital workplace ethnography to both enhance data collection and/or to reduce the number of hours the researcher spends on-site. These disadvantages of MIME could be mitigated by future researchers if remote methods were adopted in advance (rather than in response to a crisis) and if hospitals were not in crisis due to COVID-19 at the time of data collection.

### Challenge of Remote Communication

Despite the noted advantages of remote methods and their suitability in a pandemic context, there were also some clear concerns. Despite the rich insights we feel we have generated into the working lives of hospital doctors, there is an awareness that some dimensions have been lost, a sense that we are missing out by not *‘being there’* (Howlett, 2021) alongside respondents at work in the hospital. In remote communication, occasional misunderstandings are likely and clarification a little more complex to obtain, even if the WhatsApp conversation is happening in real time. Respondents explained their workspace, their working conditions to the researchers, but without visual cues, the context was sometimes unclear. This occasionally lent itself to stilted communication between respondents and researcher, most frequently illustrated by a single word, yes/no response. Occasionally opting for yes/no responses may signify that the respondent is in absolute agreement with the question or may simply be the communication style of that respondent. It may also stem from the fact that many respondents, as clinicians, are more familiar with quantitative surveys than qualitative interviews. Alternatively, it could be that one-word responses (or even no word or emoji responses) are more commonplace in a WhatsApp communication than in face-to-face communication. In these instances, the research team felt the absence of non-verbal cues from respondents and our inability to ‘see’ the context or the place from which they sent us the WhatsApp messages. Fortunately, these misunderstandings were rare during the fieldwork.

## Discussion

Qualitative research data can be used to generate invaluable insights into the everyday lived experience of health workers and can help us to see *‘the human being behind every healthcare worker’* (Kuhlmann et al., 2020). We have demonstrated the value of remote qualitative methods in rapidly generating qualitative insights. Remote qualitative methods were particularly useful during a pandemic context when in-person data collection was impossible and hospitals were ‘out of bounds’ to the research team.

In the case outlined, we adopted remote methods in an emergency (the COVID-19 pandemic) and originally envisaged it as an expedient means of continuing the research during the pandemic. However, our experiences, particularly of MIME, show that it may have merit outside of the pandemic context, either when researching hospital doctors, or other difficult-to-reach populations. As a method, MIME has potential, not only in emergency situations, but also to complement face-to-face methods, perhaps by building hybridity into research projects at the planning stage. We have found that MIME enabled respondents and researchers to conduct conversation within a digital space, which could complement in-person ethnographic data collection by providing respondents a safe space within which to share insights and experiences that they may not feel comfortable sharing in person. As Posthill explains, *‘remote fieldwork is more than a remedial measure, a “second best” choice for anthropologists unable to reach their field sites . . . It often helps us to observe familiar people and things from a different perspective, thereby creating a richer engagement with the worlds of our research respondents’* (Posthill, 2016).

We envisage that MIME could be an important and complementary method for health workforce or health services researchers in non-pandemic times. We feel that adapting to remote methods of data collection and adopting innovative ways of doing so (MIME), were a significant achievement, not least because it enabled us to continue to meet the expectations of our funders and to generate invaluable insights into the medical workforce during the COVID-19 pandemic. In much the same way that hybrid models of working, blended ways of learning will likely become the norm in the aftermath of COVID-19, perhaps hybrid ways of data collection which enable researchers to adapt the best of both worlds, will also become the norm. We show how remote methods can be very user-friendly for research respondents, especially busy professionals, allowing them some agency over both when and how much to cooperate/disclose. We also reveal from our vignettes, significant learning points for researchers conducting research with remote methods in terms of: scheduling and balancing research work (including allowing for lengthy recruitment and access issues): the importance of clear communication policies; the need to develop ethical, safe and practical working practices; ensuring researchers are supported and debriefed if they encounter traumatic or emotionally difficult content, particularly whilst working from home. The British Psychological Society (bps.org.uk) has recently published guidance on how important it is to protect people who are exposed to ‘traumatising conversations’, from ‘images’ or ‘written or auditory material’ undertaken in the home so as to avoid ‘secondary trauma’ (The British Psychological Society, 2020). Our vignettes show how troubling data encountered remotely can also unsettle and distress the recipients/researchers. Overall, our experiences also underscored the importance of ensuring realistic workloads, regular team meetings, and establishing working etiquette or protocols for unsocial hours (time in lieu) for researchers. Whilst these are part of normal face-to-face research management processes they all became even more essential and accentuated when face-to-face team meetings were restricted.

We have also shown that qualitative findings can be communicated to a wide audience in a short time-frame, which is important in a time-critical situation, such as the COVID-19 pandemic, where data can inform policy responses. This was an important driver of our research and of the decision to continue our research despite the pandemic. We felt that to support the health workforce during and after the COVID-19 pandemic, policy makers and politicians needed to be in-tune with the needs of the medical workforce and to understand how the additional pressures on the health system had impacted on individual hospital doctors. We disseminated our research findings to academic audiences Byrne J-P, Creese J, Matthews A, McDermott AM, Costello RW, Humphries N. ‘…the way it was staffed during COVID is the way it should be staffed in real life…’: a qualitative study of the impact of COVID-19 on the working conditions of junior hospital doctors. BMJ Open. 2021;11(8):e050358.Byrne et al.; Creese, Byrne, Conway, et al., 2021; Niamh Humphries et al., 2021), to the general public (Humphries, 2021; Humphries et al., 2021) and directly to policy makers. We did this to ensure that issues of doctor wellbeing, doctor retention and medical staffing remained on the policy agenda throughout the COVID-19 pandemic and also that they were informed by up-to-date information from hospital doctors.

In presenting our experience with remote qualitative methods during the COVID-19 pandemic 2020/21, we hope to inspire and guide other qualitative research teams who may need to adapt their fieldwork to unforeseen circumstances; as well as those keen to embrace hybrid models of data collection. We present Table 3; our top tips for researchers hoping to adopt remote ethnographic methods.

**Table 3.** Top Tips for Remote Ethnographic Methods.

Top Tips for remote ethnographic methods
Everyday project management
1. Ensure that researchers have a separate phone for data collection (i.e. researchers should not use their personal phones for data collection)
2. Host consent forms online (via survey monkey) so that they can be completed remotely
3. For optimal data quality and to improve rapport, researchers should react to MIME messages as they are received (if possible). This requires flexibility from researchers and also from their employers (to enable time off in lieu)
4. Do not underestimate the time required for data collection simply because it is conducted online (WhatsApp/Zoom) and, as a result sometimes doesn’t feel like ‘real’ work. Make sure to manage your workload so that you have sufficient time for data collection
Engaging with respondents
5. Allow sufficient time and energy to negotiate access and to recruit (it takes time!)
6. Ensure that respondents receive details of formal/informal support structures that they access (if needed) at any time. Ideally include these in the information leaflet
7. An interview before and after the MIME process is invaluable, the first interview to begin to get to know the respondent; the final interview to wrap up the process and to ‘debrief’ and ensure that respondents are okay with all that has been discussed during MIME
Team working and team wellbeing
8. It’s important that researchers have strong formal and informal support infrastructures in-place that they can draw on, if, and when necessary (including regular meetings for de-brief and researcher support)
9. Regular research team meetings to discuss data collection and initial findings are valuable and are a precursor to analysis

## Limitations

Beyond reassuring the reader that we received high quality respondent engagement in MIME, we have not presented detail on the content or type of responses received via MIME process, or differentiated between the type of engagement received from different types of respondents (e.g. junior vs. senior, urban vs. rural respondents) in this article. Additional detail about the data collected and further information about engagement from different groups of respondents will be presented in future articles from the MIME study. That the data presented is largely negative could be perceived as a limitation of this article and/or the MIME method. Unfortunately, this is in line with previous research on hospital doctors in the Irish health system both before and during the COVID-19 pandemic and reflects the fact, recognised internationally (OECD, 2019), that the Irish health system is under-performing and is under considerable strain.
